# Association between *OGG1* Ser326Cys polymorphism and risk of upper aero-digestive tract and gastrointestinal cancers: a meta-analysis

**DOI:** 10.1186/s40064-016-1858-5

**Published:** 2016-02-29

**Authors:** Sambuddha Das, Sayantan Nath, Aditi Bhowmik, Sankar Kumar Ghosh, Yashmin Choudhury

**Affiliations:** Department of Biotechnology, Assam University, Silchar, 788011 India

**Keywords:** Gastrointestinal, Meta-analysis, *OGG1*, Upper aero-digestive tract

## Abstract

Cancers of the upper aero-digestive and gastrointestinal tract are one of the major causes of mortality around the world. DNA repair genes play a vital role in preventing carcinogenesis by maintaining genomic integrity. Polymorphisms in the nucleotide sequence of DNA repair genes are often reported to be associated with an increased risk for different cancers. The *OGG1* gene encodes the enzyme 8-oxoguanine DNA glycosylase which removes oxidatively damaged bases of DNA. Several studies report that the *OGG1* Ser326Cys polymorphism increases the risk for cancers of the upper aero-digestive and gastrointestinal tract. However, other studies provide evidence that such an association does not exist. A meta-analysis to assess the role of *OGG1* Ser326Cys polymorphism in the cancers of the upper aero-digestive and gastrointestinal tract was therefore undertaken in order to resolve this ambiguity. Seventeen studies were recruited for this meta-analysis after screening 58 articles with a total of 5533 cases and 6834 controls for which the odds ratio with 95 % confidence interval was calculated. Begg’s funnel test and Egger’s test were performed for calculating publication bias. Our study reveals an association between *OGG1* Ser326Cys polymorphism and cancer susceptibility of the upper aero-digestive and gastrointestinal tract (CG + GG vs CC; odds ratio, OR 1.22; 95 % CI 1.05–1.41; GG vs CG + CC; OR 1.36; 95 % CI 1.09–1.70; GG vs CC; OR 1.46; 95 % CI 1.12–1.92). Subgroup analysis based on cancer types and ethnicity also revealed the association of *OGG1* Ser326Cys polymorphism to the risk for upper aero-digestive and gastrointestinal tract cancers among both the Asian and the Caucasian populations. No risk was however observed for smoking habits and *OGG1* Ser326Cys polymorphism. In conclusion, *OGG1* Ser326Cys polymorphism may be associated with the increased risk for aero-digestive tract and gastro-intestinal cancers in both Asian and Caucasian populations.

## Background

Cancer is a multifarious disease characterised by abrupt growth of cells resulting in abnormal regulation of cell-cycle progression and division (Sawyers 2004). The upper aero-digestive tract (UADT) cancers, comprising cancers of the oral cavity, pharynx, larynx and esophagus, are amongst the most common cancers globally, accounting for nearly 1 million new cases and 700,000 deaths each year (Babron et al. 2014). Gastrointestinal tract (GI) cancers which encompasses the cancers in the digestive system including gastric cancer, colorectal cancer, esophageal cancer and pancreatic cancer present an interesting pattern in distribution over the world with major occurrences in Asia (Pourhoseingholi et al. 2015). Both genetic and environmental factors contribute to the onset of cancer (Bhowmik et al. 2015). Most cancers primarily involve the dysregulation of three classes of genes viz., (proto) oncogenes, tumor suppressor genes and DNA repair genes. Such dysregulation collectively contribute to the development of cancer genotype and phenotype, which resists the natural and inherent death mechanism(s) embedded in cells (apoptosis and like processes), coupled with impairment of cell proliferation events (Bhatt et al. 2010).

DNA damage is a major perpetrator of cancer, occurring by various mechanisms, including the effect of free radicals generated through endogenous cellular metabolism or the exposure to exogenous toxins such as environmental mutagens and many carcinogens (Hoeijmakers 2009; Zhou et al. 2015). Specific genes of some important pathways such as base excision repair (BER), mismatch repair (MMR), nucleotide excision repair (NER) and double-strand break repair (DSBR) may counteract damage to DNA, thereby maintaining genomic integrity and preventing carcinogenesis (Sedelnikova et al. 2010) Oxidation of guanine, adenine and thymine accounts for the most important damage to DNA (Zhou et al. 2015). The 7,8-dihydro-8-oxoguanine (8-oxoG) is the most recurrent DNA modification generated by the oxidation of guanine leading to mispairing with cytosine and adenine during DNA replication, thereby accumulating GC to TA mutations (Bravard et al. 2009). Tobacco smoke contains various carcinogens among which benzo[a]pyrene induces 8-oxoG formation in animal tissue. Increased levels of 8-oxoG have been reported in the lung DNA of smokers in comparison to those of non-smokers, indicating a clear correlation with tobacco related carcinogenesis. A 50 % increase of 8-oxoG has also been reported in the urine of smokers compared to that of non-smokers. Studies have also found that aqueous smoke solutions can lead to the formation of superoxides and hydroxyl radicals. Together the tobacco smoke and its aqueous solution can lead to various aerodigestive and upperdigestive tract cancers (Elahi et al. 2002). The human 8-oxoguanine DNA glycosylase (OGG1), encoded by the *OGG1* gene localized on chromosome 3p25 has both DNA glycosylase and apurinic or apyrimidinic (AP) lyase activities. It removes the 8-oxoG lesion by slicing the glycosydic bond between the modified base and the sugar moiety, leaving an abasic apurinic/apyrimidinic (AP) site in DNA (Zhou et al. 2015).

There are at least 20 validated sequence variants of *OGG1* gene, of which the most studied functional polymorphism is an amino acid substitution of serine (Ser) with cysteine (Cys) (Ser326Cys) resulting from a C to G transversion at position 1245 in exon 7 of the *OGG1* gene (Bravard et al. 2009). Numerous studies pertaining to the association of this polymorphism with the increased risk for several cancers have been performed. In most of these studies the Ser326Cys polymorphism was found to increase the risk for different cancers such as head and neck cancer, colorectal cancer and gall bladder cancer (Kumar et al. 2011; Canbay et al. 2011; Srivastava et al. 2009), in association with etiological habits such as smoking (Elahi et al. 2002). However, some recent studies have also reported no association of Ser326Cys polymorphism with the increased risk for cancer (Gorgens et al. 2007; Laantri et al. 2011; Sameer et al. 2012). It has also been reported that individuals with the homozygous recessive allele of *OGG1* (^Cys^326^Cys^) and a 50 % increase in vegetable and fruit intake are at 50 % decreased risk of developing lung cancer. However, such a decrease in risk was not observed for the other genotypes (Sorensen et al. 2006). This study has therefore been undertaken in order to resolve the ambiguity regarding the association between OGG1 Ser326Cys polymorphism and susceptibility to upper aero-digestive tract and gastrointestinal cancers.

## Methods

### Literature search

Research articles relevant to the study were searched through the search engines “PubMed”, “OMIM” and “Google Scholar” using search terms like “*OGG1*, *hOGG1*”, “polymorphism, alleles, Ser326Cys, variants”, “cancer, gastric cancer, colorectal cancer, head and neck cancer, oral cancer, aero-digestive tract cancer, pharyngeal cancer, pancreatic cancer, gallbladder cancer, cancer of digestive tract, esophageal cancer.

### Selection criteria

Articles for the meta-analysis were selected if they met the following criteria: (1) Studies not prior to 2007 (2) case–control study related to the risk of *OGG1* Ser326Cys polymorphism (3) articles written in English (4) studies in which full information about genotype distributions are reported (5) Studies in which genotype distribution of control populations are in accordance with Hardy–Weinberg Equilibrium (P > 0.05) (6) only original research articles excluding reviews, letters and case reports. Studies prior to 2007 were excluded because they were mostly constrained to small study size.

### Data extraction

All the data were extracted by two investigators (Das S and Nath S) carefully and independently to maintain accuracy of the data, based on the inclusion criteria above. The information collected from each study are: author’s name, year of publication, number of cases and controls, information of the genotypes of the cases and controls, ethnicity of the study population. The study populations were divided into Asians and Caucasians based on their ethnicity. Based on the location of cancer we have defined two cancer groups as UADT cancer (which includes cancer of head and neck, oral, pharynx and larynx) and GI cancer (which includes cancer of esophagus, pancreas, gallbladder, colon and rectum). In order to study the gene environment interaction, sub group analysis was performed with smoking habits between studies. The analysis was conducted on the basis of non-smokers versus smokers in the cancer patients.

### Statistical analysis

Chi square test for Hardy–Weinberg equilibrium was performed in control populations. A P value greater than 0.05 was considered to be in accordance with Hardy–Weinberg equilibrium. Unconditional logistic regression was used to determine the odds ratio (OR) with 95 % confidence interval for all the studies. Cochran’s Q statistic (Cochran 1950) was used to find out heterogeneity across studies which was considered significant for P < 0.05. In the presence of significant heterogeneity the random effect model (DerSiminian and Laird method) (DerSimonian and Laird 1986) was used to calculate the pooled OR otherwise the fixed effect model (Mantel–Haenszel method) (Mantel and Haenszel 1959) was used. The Begg’s funnel plot and Egger’s test were used to determine publication bias (Stuck et al. 1998; Egger et al. 1997). All the analyses were performed using StatsDirect statistical software (Version 2.7.2).

## Results

### Summary of included studies

**Fig. 1:**
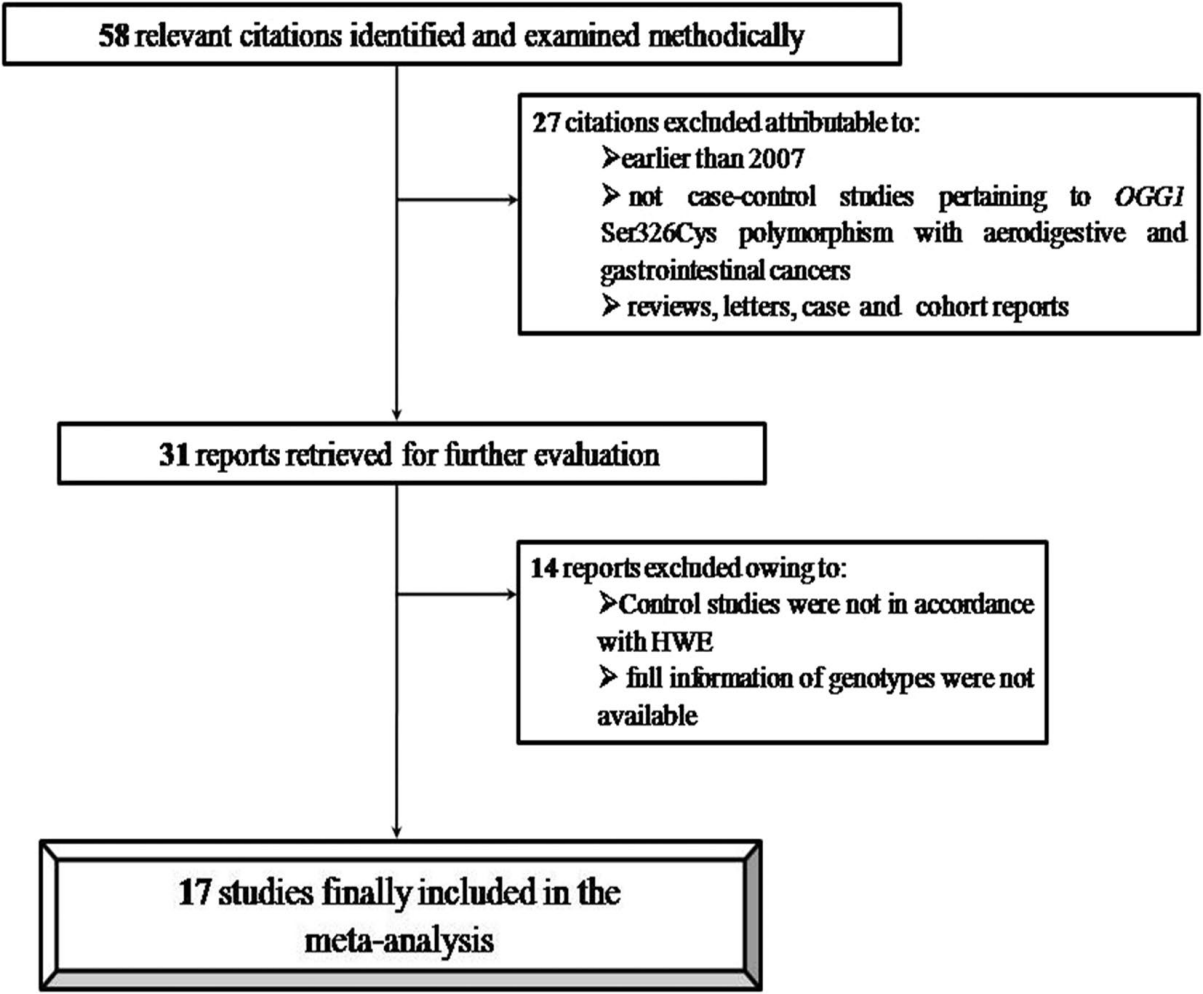
Flow chart showing the procedure of literature selection

In a preliminary search, we identified 58 research articles related to *OGG1* Ser326Cys polymorphism and the risk of head and neck, oral, pancreatic, gallbladder, colorectal and gastric cancers. Among all the articles identified, only 31 were subjected to further examination out of which only 17 (Kumar et al. 2011; Canbay et al. 2011; Srivastava et al. 2009; Gorgens et al. 2007; Laantri et al. 2011; Sameer et al. 2012; Sliwinski et al. 2011; Li et al. 2013; Upadhyay et al. 2010; Canbay et al. 2010; Palli et al. 2010; Engin et al. 2011; Curtin et al. 2009; Przybylowska et al. 2013; Pardini et al. 2008; Li et al. 2009; Srivastava et al. 2010) were found to meet the inclusion criteria (Table 1). Many of them were removed from the study due to lack of detailed information of all the genotypes and also due to non accordance of the controls with the Hardy–Weinberg equilibrium. The flowchart of the procedure for selecting the research articles for the study is described in Fig. 1.

**Table 1:** Characteristics of the studies recruited for the meta-analysis

Serial no.	Publications	Number of controls/cases	Cancer types	Country	Ethnicity	Genotyping method	Association/risk
1	Kumar et al. (2011)	278/278	Head and neck	India	Asian	PCR–RFLP	High association/increased risk
2	Sliwinski et al. (2011)	280/265	Head and neck	Poland	Caucasian	PCR–RFLP	High association/increased risk
3	Gorgens et al. (2007)	30/29	Oral	Germany	Caucasian	PCR-Cycle sequencing	No association
4	Li et al. (2013)	300/231	Nasopharyngeal	China	Asian	PCR-CTPP	No association
5	Laantri et al. (2011)	506/541	Nasopharyngeal	North-African	Caucasian	TaqMan	No association
6	Upadhyay et al. (2010)	195/135	Esophageal	India	Asian	PCR-CTPP	No association
7	Canbay et al. (2010)	247/40	Gastric	Turkey	Caucasian	PCR–RFLP	No association
8	Palli et al. (2010)	545/304	Gastric	Italy	Caucasian	TaqMan	No association
9	Engin et al. (2011)	116/106	Gastric	Turkey	Caucasian	PCR–RFLP	No association
10	Canbay et al. (2011)	247/79	Colorectal	Turkey	Caucasian	PCR–RFLP	High association/increased risk
11	Curtin et al. (2009)	1951/1582	Colorectal	USA	Caucasian	TaqMan	Weak association slightly increased risk
12	Sameer et al. (2012)	200/114	Colorectal	India	Asian	PCR-CTPP	No association
13	Przybylowska et al. (2013)	200/172	Colorectal	Poland	Caucasian	PCR–RFLP	High association/increased risk
14	Pardini et al. (2008)	532/532	Colorectal	CzechRepublic	Caucasian	PCR–RFLP	Weak association/slightly increased risk
15	Li et al. (2009)	773/722	Pancreas	USA	Caucasian	TaqMan	Weak association/increased risk
16	Srivastava et al. (2009)	204/173	Gallbladder	India	Asian	PCR–RFLP	High association/increased risk
17	Srivastava et al. (2010)	230/230	Gallbladder	India	Asian	PCR–RFLP	High association/increased risk

### Meta-analysis result

**Fig. 2:**
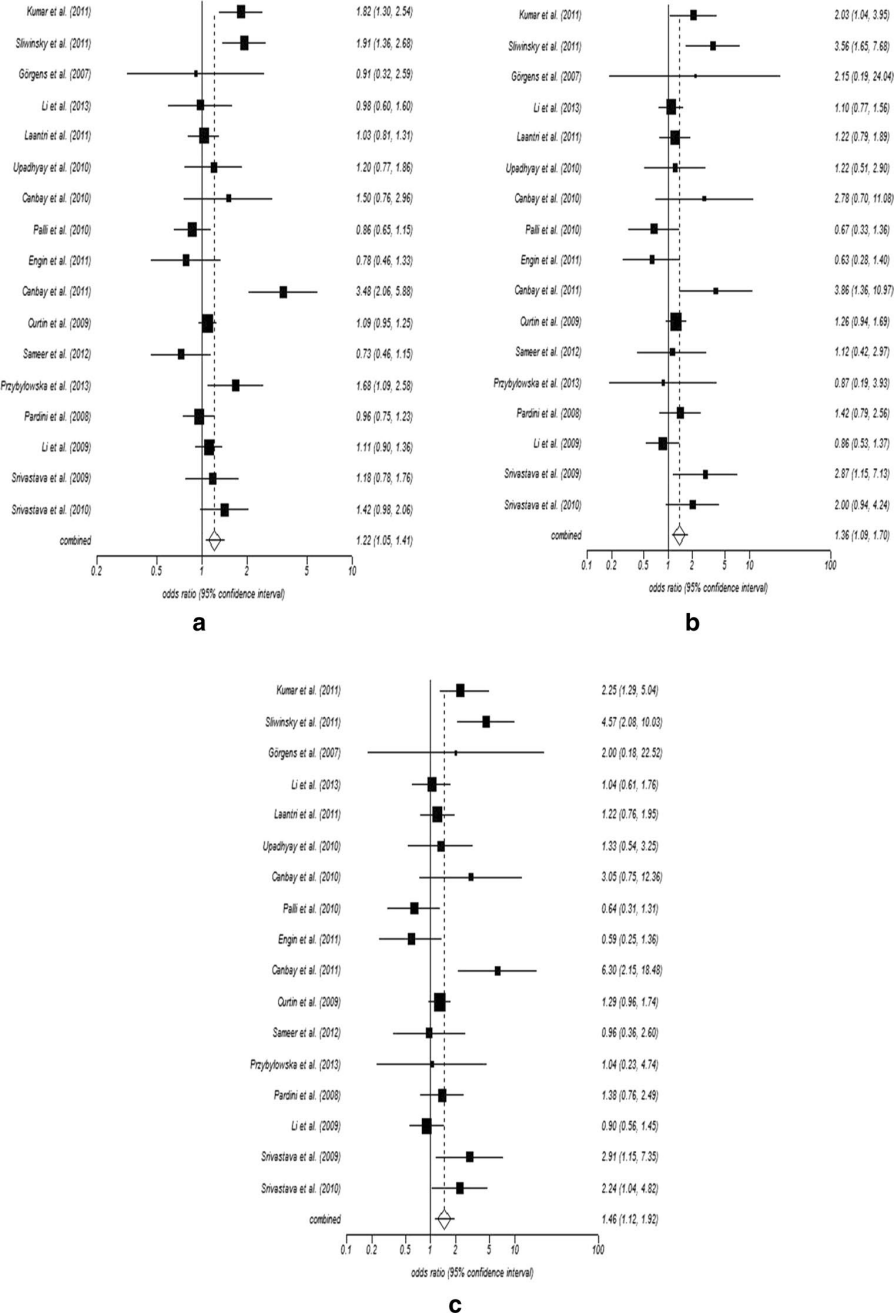
Figure showing forest plots of *OGG1* Ser326Cys polymorphism in association with upper aero-digestive tract cancer and gastro-intestinal cancer for all the three models: **a** dominant model, CG + GG versus CC, **b** recessive model, CG + CC versus GG, **c** homozygous model, GG versus CC

The meta-analysis suggests that there was significant risk for all the three models of *OGG1* Ser326Cys polymorphism (for dominant model CG + GG vs CC; odds ratio, OR 1.22; 95 % CI 1.05–1.41, recessive model GG vs CG + CC; OR 1.36; 95 % CI 1.09–1.70, homozygote comparison GG vs CC; OR 1.46; 95 % CI 1.12–1.92) (Fig. 2). Furthermore we have stratified the studies based on ethnicity to determine the role of *OGG1* Ser326Cys polymorphism in the risk for cancer among different ethnic groups (Table 2).

**Table 2:** Pooled OR of the *OGG1* Ser326Cys polymorphism based on ethnicity, sample size and cancer type

Variables	No. of studies	Case/control	Dominant model		Recessive model		Homozygous model	
			OR (95 % CI)	P^a^	OR (95 % CI)	P^a^	OR (95 % CI)	P^a^
Ethnicity								
Asian	6	1161/1407	1.21 (0.93–1.56)	0.15	1.40 (1.09–1.80)	0.009*	1.56 (1.15–2.11)	0.004*
Caucasian	11	4372/5427	1.22 (1.01–1.48)	0.03*	1.29 (0.95–1.76)	0.104	1.40 (0.97–2.03)	0.07
Sample size								
<500	8	943/1474	1.31 (0.93–1.85)	0.122	1.64 (1.14–2.35)	0.007*	1.83 (1.01–3.29)	0.044*
≥500	9	4590/5360	1.16 (0.99–1.35)	0.06	1.23 (1.05–1.44)	0.012*	1.32 (0.99–1.76)	0.06
Cancer type								
GI	11	4054/5245	1.17 (0.97–1.40)	0.09	1.28 (0.95–1.74)	0.103	1.36 (0.96–1.92)	0.08
UADT	6	1479/1589	1.32 (1–1.74)	<0.05*	1.37 (1.09–1.73)	<0.05*	1.74 (1.08–2.80)	0.02*

^a^P heterogeneity

* Significant at P < 0.05

**Fig. 3:**
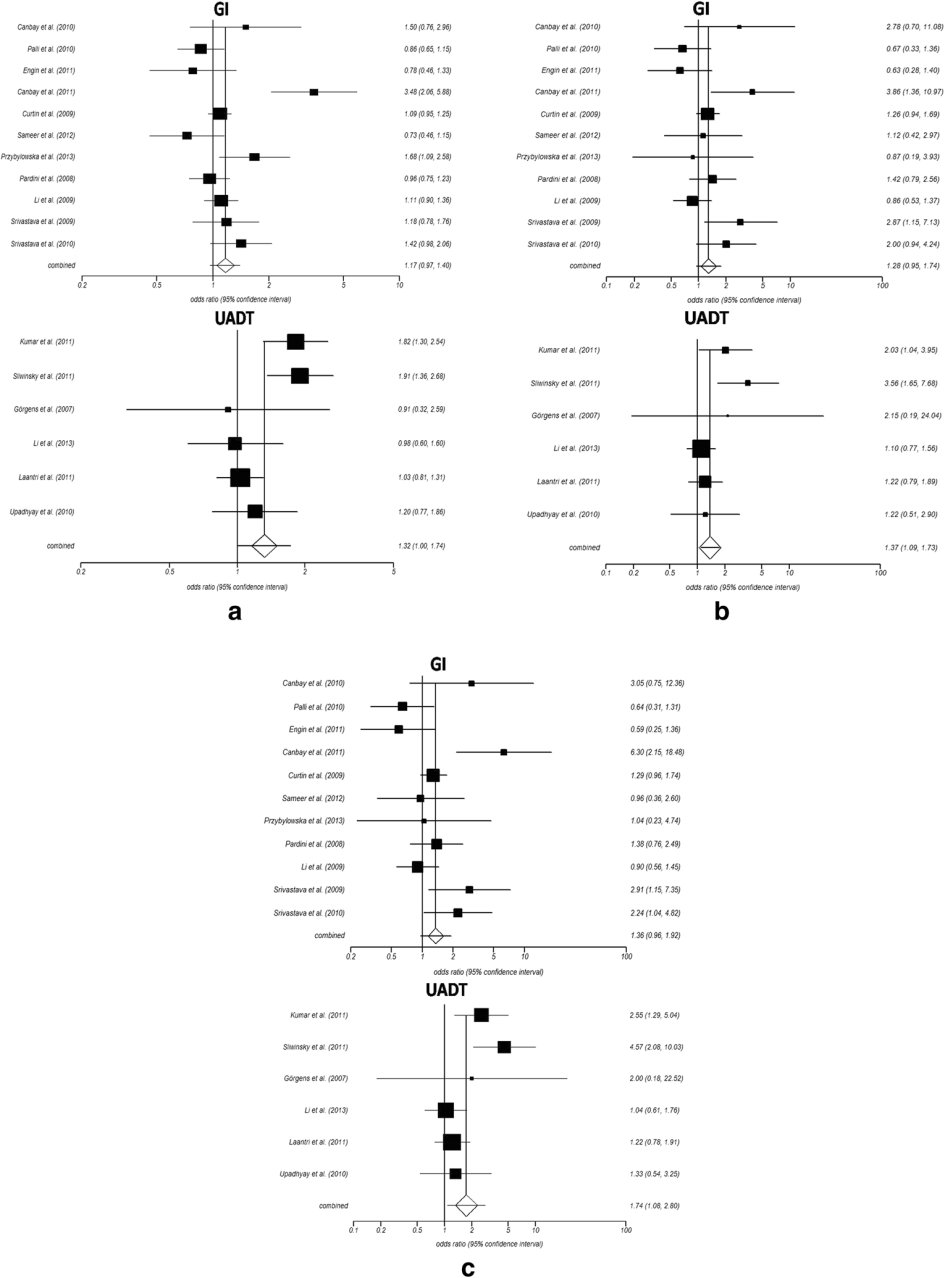
Figure showing forest plot of stratified analysis based on cancer types i.e. gastro-intestinal (GI) and upper aero-digestive tract (UADT) cancer in association with *OGG1* Ser326Cys polymorphism for all the three models: **a** dominant model, CG + GG versus CC, **b** recessive model, CG + CC versus GG, **c** homozygous model, GG versus CC

The frequency of Ser/Cys and Cys/Cys genotype was slightly higher among cancer cases (0.37 and 0.06) than among the controls (0.35 and 0.05) for the Caucasian population. A similar trend was also observed in the Asian population where the frequency of Ser/Cys and Cys/Cys was higher among the cases (0.43 and 0.15) than the controls (0.41 and 0.12). Results suggest that the polymorphism was a risk for cancer among both the Asian (CG + GG vs CC; OR 1.21; 95 % CI 0.93–1.56, GG vs CG + CC; OR 1.40; 95 % CI 1.09–1.80, GG vs CC; OR 1.56; 95 % CI 1.15–2.11) and the Caucasian (CG + GG vs CC; OR 1.22; 95 % CI 1.01–1.48, GG vs CG + CC; OR 1.29; 95 % CI 0.95–1.76, GG vs CC; OR 1.40; 95 % CI 0.97–2.03) populations for all the three models. Stratification on the basis of cancer types reveals that there was an elevated risk for individuals with this polymorphism for both the GI (CG + GG vs CC; OR 1.17; 95 % CI 0.97–1.40, GG vs CG + CC; OR 1.28; 95 % CI 0.95–1.74, GG vs CC; OR 1.36; 95 % CI 0.96–1.92) and UADT (CG + GG vs CC; OR 1.32; 95 % CI 1–1.74, GG vs CG + CC; OR 1.37; 95 % CI 1.09–1.73, GG vs CC; OR 1.74; 95 % CI 1.08–2.80) cancer (Fig. 3). However the risk for GI cancer was not significant.

### Smoking habit and *OGG1* polymorphism

Data pertaining to the habit of smoking was available only for a few of the included studies and the genotypes of *OGG1* Ser326Cys stratified on the basis of smoking habit was also available only for cancer cases and not for the controls. We therefore conducted a meta-analysis with only 6 studies in order to determine the association of the polymorphism with the occurrence of GI and UADT cancers in patients who smoked compared to those who did not smoke. The interaction between *OGG1* polymorphism and smoking habit was not found to increase the risk for cancer in all the three models (dominant CG + GG vs CC; OR 0.96; 95 % CI 0.71–1.30, recessive GG vs CG + CC; OR 1.06; 95 % CI 0.69–1.62, homozygote GG vs CC; OR 0.97; 95 % CI 0.58–1.62).

### *OGG1* polymorphism and risk of different types of cancer

We observed that the cancers categorized in this study as GI and UADT cancers were of different types, viz. head and neck cancer, oral cancer, nasopharyngeal cancer, esophageal cancer, gastric cancer, colorectal cancer, pancreas cancer and gall bladder cancer (Table 3). Oral cancer, nasopharyngeal cancer and esophageal cancer are grouped together alongwith head and neck cancer (Barnes 2005). For head and neck cancer a very significantly increased risk was observed for all the three models (CG + GG vs CC; OR 1.3; 95 % CI 0.99–1.73, GG vs CG + CC; OR 1.37; 95 % CI 1.09–1.73, GG vs CC; OR 1.69; 95 % CI 1.06–2.68). Similarly, increased risk was also observed for colorectal cancer for all the models (CG + GG vs CC; OR 1.29; 95 % CI 0.9–1.84, GG vs CG + CC; OR 1.34; 95 % CI 1.05–1.71, GG vs CC; OR 1.38; 95 % CI 1.08–1.77). However, for gastric cancer we did not observe any risk for all the three models (CG + GG vs CC; OR 0.9; 95 % CI 0.71–1.14, GG vs CG + CC; OR 0.78; 95 % CI 0.47–1.29, GG vs CC; OR 0.76; 95 % CI 0.45–1.27). Meta-analysis could not be performed for pancreas cancer and gall bladder cancer due to less number of studies meeting the selection criteria (<3).

**Table 3:** Pooled OR of the *OGG1* Ser326Cys polymorphism based on different types of cancer

Type of cancer	No. of studies	Case/Control	Dominant model		Recessive model		Homozygous model	
			OR (95 % CI)	P^a^	OR (95 % CI)	P^a^	OR (95 % CI)	P^a^
Head and neck	6	1479/1589	1.3 (0.99–1.73)	<0.05*	1.37 (1.09–1.73)	0.007*	1.69 (1.06–2.68)	0.02*
Gastric	3	450/908	0.9 (0.71–1.14)	0.39	0.78 (0.47–1.29)	0.34	0.76 (0.45–1.27)	0.3
Colorectal	5	2479/3130	1.29 (0.9–1.84)	0.15	1.34 (1.05–1.71)	0.01*	1.38 (1.08–1.77)	0.009*

^a^P heterogeneity

* Significant at P < 0.05

### Heterogeneity test

In this study significant heterogeneity was observed for all the models of *OGG1* Ser326Cys polymorphism (CG + GG vs CC; P < 0.0001, *I*^*2*^ = 69.1 %, GG vs CG + CC; P = 0.0248, *I*^*2*^ = 44.4 %, CC vs GG; P = 0.0019, *I*^*2*^ = 57.1 %) and subsequently the random effect model was selected. We then tried to assess the source of heterogeneity based on ethnicity (Asian and Caucasian), sample size (<500 subjects and ≥500 subjects) and cancer types (GI cancer and UADT cancer). From the analysis it was found that subsequent heterogeneity was contributed by Caucasian population (*I*^*2*^ = 74.3 %) and sample size of below 500 (*I*^*2*^ = 72.6 %) among the studies. While in case of cancer types heterogeneity was found to be contributed by both the GI (*I*^*2*^ = 70.3 %) and UADT (*I*^*2*^ = 64.9 %) cancers.

### Publication bias and sensitivity test

**Fig. 4:**
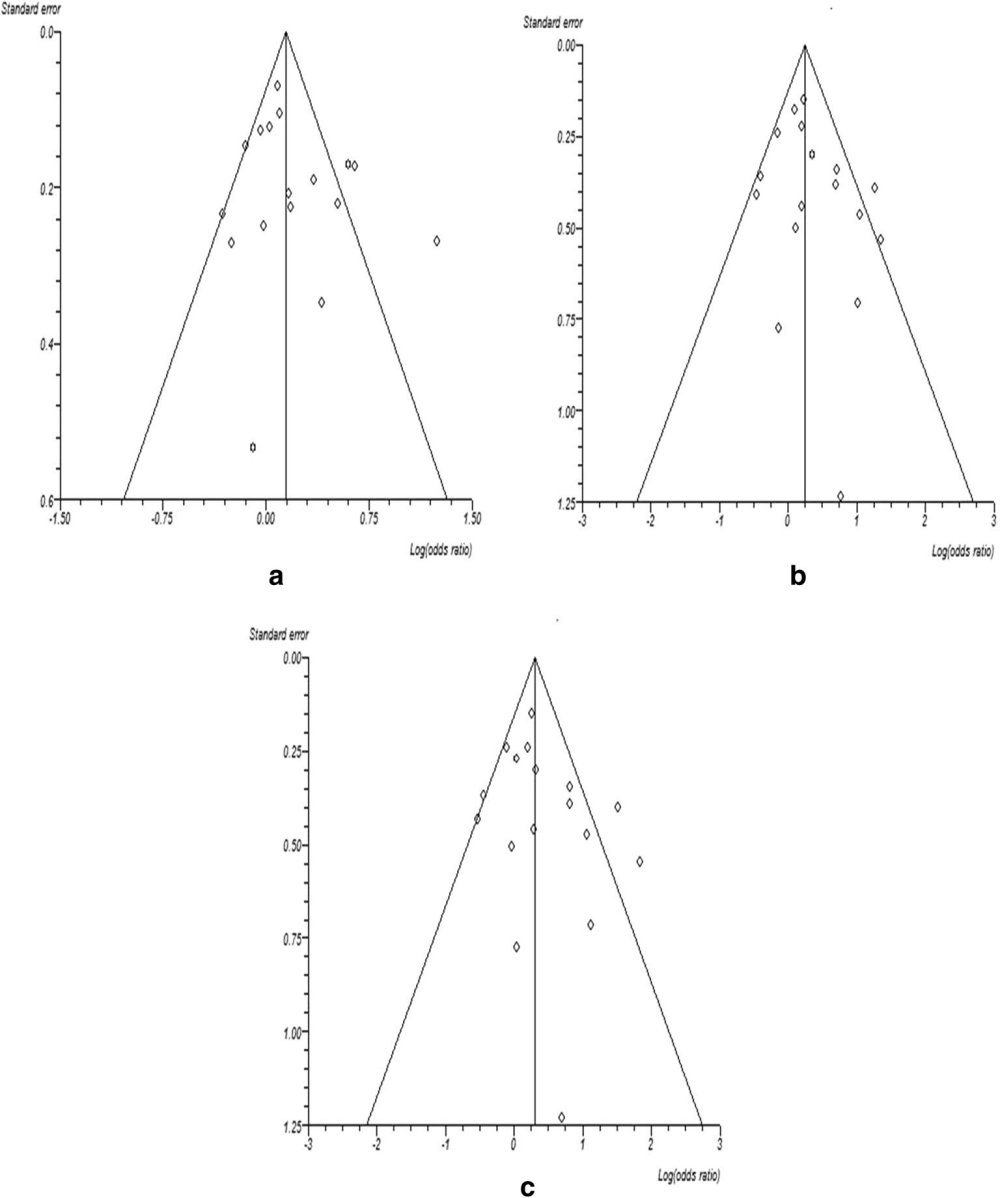
Figure showing funnel plot for publication bias **a** dominant model, CG + GG versus CC, **b** recessive model, CG + CC versus GG, **c** homozygous model, GG versus CC

Begg’s funnel plot (Fig. 4) and Egger’s test were used to determine publication bias. We did not find any asymmetry in the funnel plot indicating no publication bias. The result was validated by the Egger’s test for all the models (p > 0.05). Sensitivity test was performed in order to find out the effect of individual study on the pooled odds ratio. From the sensitivity test it was observed that no individual study was found to significantly impact on the overall result.

## Discussion

DNA repair genes play an important role in maintaining the genomic integrity of a cell. Polymorphisms in these genes may alter the protein function and thus hamper the DNA repair capacity of an individual (Goode et al. 2002). The *OGG1*gene encodes the enzyme required for removal of the 8-oxoG adduct from DNA. Although many polymorphisms have been reported in *OGG1,* the C/G polymorphism at the 326 codon of exon 7 which results in an amino acid substitution from serine to cysteine is of great importance as it has been linked to the increased risk for different cancers. In few studies, the OGG1 protein encoded by the 326^Ser^ variant is found to have more DNA repair activity than that coded by the 326^Cys^ variant indicating the role of this polymorphism in carcinogenesis (Elahi et al. 2002). Previous studies including several meta-analyses pertaining to the role of *OGG1* Ser326Cys polymorphism with the risk for different cancers were found to be ambiguous. Hence, several meta-analyses were also performed in recent years in order to address this ambiguity. Meta-analyses by Yan et al. (2014), Zhang et al. (2011) and Wang et al. (2011) did not find any association between *OGG1* polymorphism and the risk for pancreatic cancer, colorectal cancer and gastric cancer respectively. However, another study by Su et al. (2014) reported that the *OGG1* polymorphism was significantly associated with increased risk for colorectal cancer in Caucasian population. Similar risk was also found for esophageal cancer in a study by Wang et al. (2013). Thus, the variations in the results among different studies still persisted. In an effort to resolve this ambiguity, we have performed a meta-analysis to determine the role of *OGG1* Ser326Cys polymorphism on the risk for UADT cancer and GI cancer which includes different independent case control studies on head and neck cancer, gastric cancer, colorectal cancer, pancreatic cancer, gallbladder cancer and esophageal cancer. Our study has revealed a significant association of *OGG1* polymorphism with UADT and GI cancer risk. For all the three models (CG + GG vs CC, GG vs CG + CC and GG vs CC) we observe a significant risk, clearly indicating the role of the mutant G allele in increasing the risk for UADT and GI cancers.

Stratified analysis based on ethnicity reveals that the *OGG1* polymorphism increases the risk for cancer in both Asian and Caucasian populations. The maximum risk for Asian population was observed for GG vs CC genotype with a 1.56 fold increase in risk. Similar trend was also observed for the Caucasian population with a 1.4 fold increase in risk. We also observed a significant increase in risk by 1.4 fold among Asians with GG vs CG + CC genotype. The 17 articles included in this study were found to have been performed on either Asian or Caucasian populations. Hence, this meta-analysis reports findings pertaining to only these two ethnicities, although the ethnicity was not a pre-determined criterion for selection.

Environmental factors such as smoking in association with different polymorphisms were found to play an important role in the onset of various cancers (Das et al. 2015). However, our study revealed no association between the habit of smoking and *OGG1* Ser326Cys polymorphism. This may be due to a decrease in sample size resulting from stratification of data based on smoking habit. Our study based on the risk for different types of cancers and *OGG1* polymorphism showed highly significant risk for both the head and neck and colorectal cancers which was in accordance with Su et al. (2014) and Wang et al. (2013) respectively. However, no risk was observed for gastric cancer. On further stratifying our study into GI and UADT cancers it was observed that individuals with G allele are at an increased risk for both the type of cancer. For both GI and UADT cancers the maximum risk was observed for the homozygote model with a 1.36 and 1.74 folds increase in risk respectively.

Heterogeneity testing revealed significant heterogeneity among all the three models in our study. However, while trying to assess the source of heterogeneity, we found that maximum heterogeneity was contributed by Caucasian population, indicating a presence of ethnicity specific effect of *OGG1* on the risk for UADT and GI cancers. Significant heterogeneity was also contributed by sample size and cancer types which indicate that the study recruited for the meta-analysis might have contributed to the increased heterogeneity for which the random effect model was used for calculating the pooled OR. Besides that we have also calculated publication bias by Begg’s funnel plot and Egger’s test, the results of which indicated no such bias in our study. This authenticates that our meta-analysis results are relatively stable.

Although we have conducted our meta-analysis with a very large number of cases and controls the study has some limitations. Firstly, most of the study groups were of Caucasian origin and only few studies pertaining to the Asian population could be recruited for the study. Secondly, we were not able to conduct our study for the other ethnicities, which should be researched in future studies; and thirdly, only a few studies recruited for the meta-analysis contained detailed information on the smoking habits of cancer patients and controls, as a result of which the analysis based on smoking habit must be further validated on a larger study group.

In conclusion, our meta-analysis provides evidence that the *OGG1* Ser326Cys polymorphism may be associated with an increased risk for UADT and GI cancers in both Asian and Caucasian populations.
